# A Hybrid Effectiveness-Implementation Study of a Multi-Component Lighting Intervention for Hospital Shift Workers

**DOI:** 10.3390/ijerph17239141

**Published:** 2020-12-07

**Authors:** Elizabeth M. Harrison, Emily A. Schmied, Alexandra P. Easterling, Abigail M. Yablonsky, Gena L. Glickman

**Affiliations:** 1 Center for Circadian Biology, University of California San Diego, La Jolla, CA 92093, USA; a.easterling@alumni.albany.edu (A.P.E.); gena.glickman@usuhs.edu (G.L.G.); 2 Leidos, Inc., San Diego, CA 92121, USA; 3 Health and Behavioral Sciences Department, Naval Health Research Center, San Diego, CA 92016, USA; abigail.m.yablonsky.mil@mail.mil; 4 School of Public Health, San Diego State University, San Diego, CA 92182, USA; eschmied@sdsu.edu; 5 Naval Medical Center, San Diego, CA 92134, USA; 6 Departments of Psychiatry and Neuroscience, Uniformed Services University of the Health Sciences, Bethesda, MD 20814, USA

**Keywords:** shiftwork, light, circadian, sleep, LAN, implementation

## Abstract

Simple lighting solutions may mitigate the harmful effects of shiftwork. This hybrid effectiveness–implementation study evaluated a multi-component lighting intervention in hospital nurses that included 6500 K architectural lighting in the nurses’ station plus optional behavioral components (a lightbox, blueblocker glasses, eyemasks) with instruction about appropriately timed usage. Selective improvements from baseline were observed in on-shift performance, sleep quality, and caffeine consumption in day workers (all *p* < 0.05); off-shift sleepiness scores improved for night workers (*p* < 0.05). Further, self-reported measures of quality of life improved for both groups (*p* < 0.05). Preliminary implementation data from interviews and questionnaires suggest perceived benefits and high acceptability of the intervention.

## 1. Introduction

Shiftwork causes chronic sleep loss, exposure to light at night, and circadian misalignment, with resultant deleterious effects on health and performance [1]. Shiftwork is, however, necessary to provide round-the-clock hospital care. Both phase-shifting and acute-alerting properties of light may aid shift workers [2,3], with increased light intensities and shorter wavelength energy eliciting relatively greater effects [2].

A complicating factor is that circadian-based interventions largely depend on phase, which is variable across and within shift workers. Further, in the translation of basic findings to applied settings, mitigating factors (e.g., preferences, workload) should be considered, as they may present barriers to intervention implementation and impact efficacy [4], though such data are relatively uncommon in reports of sleep and circadian interventions [5]. In short, there can be no one-size-fits-all solution. The aim of this study was to improve the sleep and circadian health of hospital shift workers via a lighting intervention, which increased the biological potency of the light exposure received in the workplace while reducing light exposure just before and during sleep. To address these issues, we took a multi-component, individually customizable approach, capitalizing on current practices in the workplace and including measures of intervention implementation.

## 2. Materials and Methods

### 2.1. Participants

Participants were 16 nurses and 3 corpsmen working 12 h shifts in the Labor and Delivery Department of the Naval Medical Center San Diego (*n* = 19 females; 9 day workers; 10 night workers; mean age ± SD = 34.74 ± 8.09 years), with 10.43 ± 9.14 years in shiftwork. Participants also reported little to no formal education on sleep or circadian rhythms before the intervention (Table S1). Participants reported corrected vision (*n* = 7) but no other vision conditions. See Table S1 in Supplementary Materials for more detailed demographic information. Participants were required to be on the same shift type for the duration of the study in order to be enrolled; there were no other exclusion criteria.

Work shifts began at 7 am or 7 pm daily and were matched to the extent possible across conditions. Nurses and corpsmen work either permanent “day” or “night” shifts, or rotate between the two approximately every 6–8 weeks. For each shift type, participants largely worked a variant of a “blocked” 2-2-3 schedule, with two days on, two days off, then three days on. Each participant was working the same shift type and, to the extent possible, the same weekly schedule in both baseline and intervention conditions (see Section 2.2).

### 2.2. Protocol

**Figure 1:**
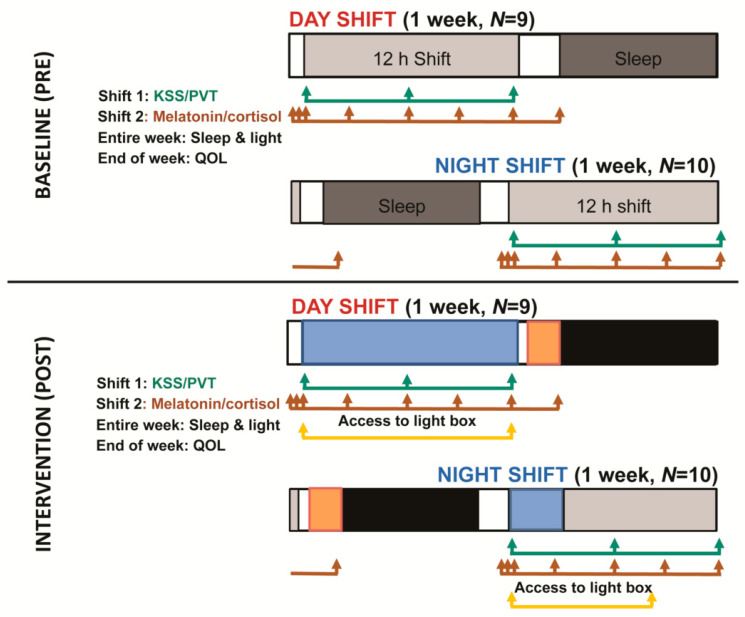
A graphic depiction of data collected during baseline and intervention weeks. When possible, alertness measures were collected on the first work day of the week and saliva sampling took place on the second work day of the week. Participants were prompted at the beginning, middle, and end of one shift to submit their sleepiness scores and perform the Psychomotor Vigilance Task (PVT). On a second shift, they collected eight saliva samples across the waking day, which were later assayed for cortisol and melatonin. Sleep and light data were collected across each week via actigraphy. At the end of each week, participants completed a questionnaire which included questions about the light as well as the quality of life measure. For the intervention period, the same data were collected. Blue boxes represent exposure to the overhead bulbs, orange boxes represent suggested times for use of blueblocking glasses, and black boxes represent times of sleep with optional use of eyemasks. Day workers were exposed to the new bulbs throughout their shift, could use the light box throughout the shift, and could wear blueblockers before, and eyemasks during, sleep. Night workers had the same protocol, with two exceptions: the new overhead lights were on only at the beginning of the shift, in keeping with the department’s standard practices (turning off lights near 10 pm for the patients), and the light box was only to be used up until 4 am (3 h before shift end). The end-of-week survey for the intervention week also included satisfaction and feasibility questions regarding the three optional components.

We utilized a hybrid effectiveness–implementation approach, assessing implementation factors alongside efficacy [6]. Procedures were conducted according to the Declaration of Helsinki and approved by the Naval Medical Center San Diego Institutional Review Board (NMCSD.2012.0002). All subjects participated in two, 7 day data collection periods (Figure 1), one just prior to the architectural light change (baseline), and one following it (intervention).

The majority of participants (*n* = 15) completed both the baseline and intervention 7-day study windows on the same calendar days, which ranged from a Sunday to a Saturday. An attempt was made to have the baseline and intervention weeks as close as possible to one another for each individual, with one washout week in between; however, for one individual, there was less than one week (3 days), and there were 2–3 weeks between conditions for three individuals. All baseline data were collected from the 11 days prior to the light change (mode = days 1–8 (*n* = 9 day and 9 night workers), and all intervention data was from the 3–24 days following it (mode = days 3–10 (*n* = 8 day and 7 night workers)). Across all subjects, the number of shifts each individual worked did not differ between baseline and intervention weeks (paired *t*-test, *p* = 0.14).

Participants were instructed on all materials and data collection procedures during the consent process. Each of the two, 7-day study windows (baseline and intervention conditions) was set for each individual prior to data collection. When possible, the day alertness was monitored (see Section 2.3) was the first in a series of two or more shifts, and salivary collection was scheduled for the subsequent shift (*n* = 18/19), though the most important determinant of which day of the week individual participants collected measures for each of the two conditions was maintaining intrapersonal consistency between schedules for the baseline and intervention weeks. Participants were instructed to notify study staff in the event of a last minute schedule change and were also encouraged to text, email, or call study staff with any questions throughout the study. These schedule changes resulted in only a few last-minute adjustments to data collection days.

At baseline, the window-less staff area was illuminated by ten fluorescent fixtures, with continuous dim recessed lighting around the periphery. Before the study, the hospital ward’s standard lighting schedule was to turn on the overhead lights above the nurses’ station near the beginning of the day shift, leave them on during the day, and to turn them off at approximately 10 pm to signal nighttime for patients. The exact timing varied somewhat as staff were allowed to turn the lights on and off at will. For the study, toggle-switch timers (AutoChron, Southwest Environmental, Inc; San Diego, CA, USA) were introduced 12 days before the study and maintained throughout the protocol to automate the ward’s standard lighting schedule (entire dayshift and the first three hours of nightshift). Under the standardized timed conditions (beginning before baseline), the lighting transitions were staggered such that half of the overhead lights came on at 6:45 am and the other half at 7:30 am; similarly, for lights off, the first half went off at 9:45 pm, and the second at 10:15 pm. Recessed lighting around the perimeter of the room remained on constantly through both conditions. In-person checks and a stationary light meter (DLM112SD, General Tools; Seacaucus, NJ, USA) confirmed timer functionality.

For the intervention, Philips 32w F32T8/TL741 bulbs (4100 K; ~62.3 µW/cm^2^; Philips, Amsterdam, The Netherlands) were replaced with the most biologically potent off the shelf, commercially-available fluorescent bulbs that were compatible with existing fixtures: the relatively cooler color temperature Sylvania 36w Activa bulbs (6500 K; ~72.6 µW/cm^2^, Sylvania, Wilmington, MA, USA). Irradiance and spectral composition were measured (IL1700, International Light Technologies; Peabody, MA, USA; Ocean Optics USB2000 spectroradiometer, Ocean Optics; Dunedin, FL, USA; for Spectral Power Distributions, see Figure S1 and [7]).

A bright (~2240 µW/cm^2^) white lightbox (Sunray II, Sunbox; Frederick, MD, USA) was made available during the intervention in a room adjoining the nurses’ station. Participants were instructed to sit 18 inches from the lightbox for 5–15 min at a time if they felt sleepy during their shifts, without gazing directly at it (outside of the last 3 h of the nightshift; Figure 1). As research indicates that the bright light encountered upon leaving the shift in the early morning is detrimental to subsequent sleep and shifts underlying phase [8], we chose to avoid what we know to be a biologically potent stimulus close to the desired bed times of some. Blue-blocking glasses (UVEXS0360X Ultra-Spec2000 SCT-Orange; Honeywell, Morris Plains, NJ, USA) and eyemasks (Meta-U, Shanghai, China) were provided for the intervention. Participants were instructed to use the blue-blocking glasses after work and before bed but not while driving, for safety [9], and encouraged to use eyemasks during sleep, including naps. Text reminders to collect data and use optional components were sent throughout the study.

### 2.3. Measures

Continuous measures of activity and photic exposure patterns were collected via actigraphs (Actiwatch-2 (*n* = 15) and Spectrum Plus (*n* = 4); Philips, Murrysville, PA, USA), and a diary tracked daily sleep and caffeine use [10]. At the beginning, middle, and end of one workshift in each condition, participants completed a validated, 3 min tablet-based Psychomotor Vigilance Task (PVT) (Pulsar Informatics, Philadelphia, PA, USA) [11] and a modified Karolinska Sleepiness Scale (KSS) (“*On a scale from 1 to 9, how sleepy are you? (1 = extremely alert, 5 = neither, 9 = fighting sleep)”)*. For the PVT, two tablets were set to a standardized angle of ~60° and affixed at standing height to the counter surrounding the nurses’ station. Participants were instructed on the use of the tablet and Joggle software during the consent process, and instructions were posted next to the tablet to serve as a reminder, if needed. Data were uploaded to a server during daily checks. KSS scores were also assessed at 7 pm on one day off. Participants collected 8 salivary samples over the course of a second work day in both conditions. The first three samples were to be completed within the first hour of waking (~wake, wake + 30m, and wake +60 min). Four samples were taken over the course of the work shift (3, 6, 9 and 12 h into the shift). Finally, one sample was taken before the main sleep period, or bedtime, following the work shift (BT). Participants were supplied with pre-labeled tubes and portable, insulated cooler bags with ice packs for storage during transport with a tag on which to note the actual times of sampling. Melatonin and cortisol were assayed via enzyme-linked immunosorbent assay (ELISA), and data were normalized as a percent of individual mean. At the end of each 7 day period, participants were prompted to complete a short 10 min online survey (Qualtrics; Provo, UT, USA) consisting of the quality of life index (QLI) and original questions about the light. The QLI has been demonstrated to have high reliability, construct validity, and internal validity, as well as to being sensitive to medical and behavioral interventions in clinical populations [12,13,14,15]. Participants rated both their satisfaction with and the importance of 33 items. One item regarding unemployment was removed for our version (as all participants were employed). Per scoring instructions, the 32 satisfaction responses were normalized, weighted by the 32 importance scores, then summed to generate one total score, as well as subscores in four domains: health and functioning, social and economic, psychological/spiritual, and family. In all cases, questionnaires were completed within 4 days of being distributed (mean ± SD = 1.43 ± 1.57 d), and baseline questionnaires were all completed before the change in lighting. As light timing and dose differed between day workers and night workers, shift types were analyzed separately with paired *t*-tests or mixed models. Measures with repeated sampling from the same subject during a single condition were analyzed using linear mixed models, with subject as a random factor and condition as a fixed factor. For variables collected during work shifts (KSS, PVT), time on shift was also included as a fixed factor (beginning, middle, end). Measures assessed with a single data point precondition, such as quality of life and off-shift KSS, were analyzed across conditions with paired *t*-tests. Effects of age on all sleep variables were assessed, and none were found in our sample; therefore, age was not included in any model.

A mixed-methods approach assessed factors that could influence intervention adoption and sustainment. Pre- and post-intervention, participants reported on experience with the overhead light (comfort, brightness, symptoms, desire to keep it). Post-intervention survey questions assessed satisfaction with the intervention, as well as the perceived ease of use and likelihood of continuing use for each component. Additionally, a subsample of intervention participants and department leaders participated in one-on-one telephone interviews. Leaders’ perceptions of the intervention and organizational factors that may affect sustained intervention usage were probed. Post-intervention, participants were asked for suggestions on how to sustain the intervention and to describe their experiences, including barriers or enablers encountered. A codebook based on the Consolidated Framework for Implementation Research (CFIR) guided the analysis of the interview transcripts [16] (Nvivo v12; QSR International, Doncaster, Victoria, Australia). Themes were categorized based on CFIR domains, and emergent subthemes were noted.

## 3. Results

### 3.1. Intervention Use

Based on daily diaries, time spent in the nurses station did not differ between conditions *(n* = 16, *t*_(15)_ = 1.32, *p* = 0.20). Three participants (2 day, 1 night) did not complete the relevant question on their diaries.

Most participants (58%) reported in the end-of-week questionnaire that they used all of the three, optional intervention pieces (lightbox (LB), blue-blocking glasses (BB), and eyemasks (EM)) at some point during the intervention week (*n* = 11; 4 day and 7 night). Six individuals chose only one or two optional components; three individuals reported only using BB and EM (2 day and 1 night); and three individuals only reported using the LB (1 day and 2 night). Only two individuals reported abstaining from all three optional components (both day). Based on the participants with complete diaries (*n* = 16), those electing to use the eyemasks and blue-blocking glasses used them daily or nearly daily throughout the intervention week. Based on the lightbox usage log, thirteen nurses reported using the lightbox between one and four times during the intervention week (*n* = 6 day, 8 night; Figure S2). Exposure duration ranged from 3 to 17 min, and averaged 6.02 min. The average cumulative duration over the intervention week was 11.69 min (see Figure S3 for an example actogram with light data). Day nurses’ use of the lightbox clustered at approximately 14:41, and night nurses at approximately 1:23. One night nurse used the lightbox after 4 am in spite of instructions not to do so (at ~4:40 am; Figure S2).

### 3.2. Effectiveness Measures

**Figure 2:**
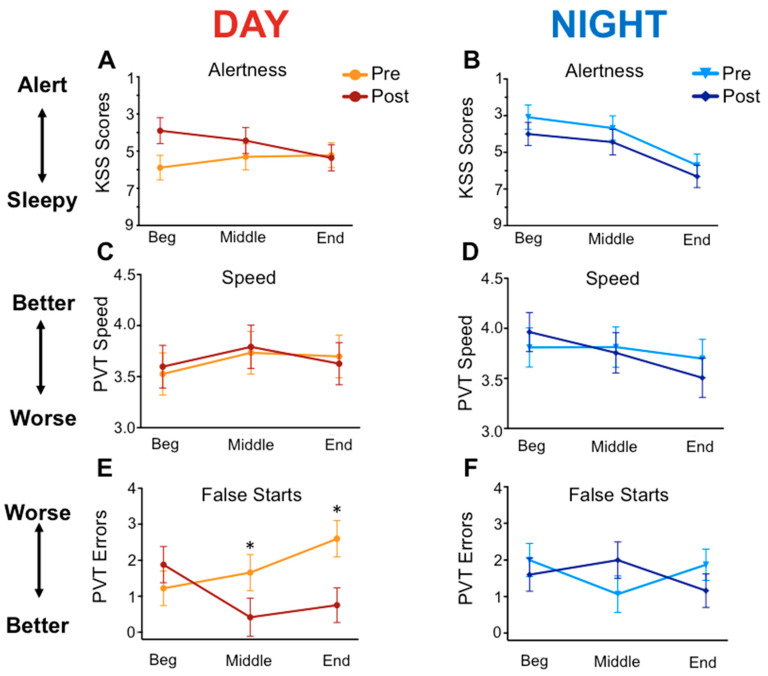
Self-reported sleepiness (KSS) and performance (PVT) on shift from day and night workers pre- and post-intervention. Participants were prompted via text at the beginning, middle, and end of one work day in each condition to report sleepiness on a scale of 1–9 (Karolinska Sleepiness Scale; KSS) and to complete a validated, 3 min tablet-based version of the PVT [11]; Pulsar Informatics, Philadelphia, PA, USA). Panels (**A**) and (**B**) are KSS scores across shift for day and night workers, respectively. Panels (**C**) and (**D**) are PVT speed, and (**E**) and (**F**) are PVT False Starts. Asterisks denote *p* < 0.05.

KSS scores varied by time on shift for night workers (*p* < 0.001), with highest values at the end of the shift (*p* < 0.001), but did not vary by condition (*p* = 0.16; Figure 2). There was a trend for lower on-shift sleepiness at the beginning of the shift during the intervention for day workers only (*p* = 0.06 for condition and *p* = 0.09 for condition × time; Figure 2A); there were no other effects for either group (all *p* > 0.16). KSS scores at 7 pm on days off were significantly lower for the intervention condition in night workers (*p* < 0.05) but not day workers (*p* = 0.37). PVT reaction time was slowest mid-shift for day workers (*p* < 0.05; Figure 2C) and did not differ by condition (*p* = 0.76); lapses followed a similar pattern (all *p* < 0.12). Fewer middle- and end-of-shift false starts were made by day workers during the intervention versus baseline (*p* < 0.01 for condition and conditionXtime; Figure 2E). No statistical differences in PVT scores were found in night workers (Figure 2D,F).

Sleep quality improved (*p* < 0.001; Table 1) and caffeine consumption was lower (*p* < 0.05) during the intervention for day workers; neither changed for night workers (both *p* > 0.33). No differences were found across conditions for either group in any other diary- or actigraphy-based sleep parameters (all *p* > 0.18; See Table 1 and Table S2). Average light profiles for all participants for both conditions are shown in Figure S4. For cortisol, effects of time of day were found for both groups (both *p* < 0.001), with no difference between conditions and no interactions (all *p* > 0.30). For melatonin, an effect of time of day was found for day workers (*p* < 0.001), but not for night (*p* = 0.40), with no condition or interaction effects (all *p* > 0.83; Figure S5). Total quality of life and the health and functioning subscale were higher in both groups during the intervention week (both *p* < 0.05; Table 1), as was the Family subscale for day workers (*p* < 0.05). All other scores showed no change (all *p* > 0.10). Effect sizes (Cohen’s *d*) from all significant QLI comparisons are moderate (>0.51) or small to moderate (>0.34) and within the range of score change reported by other medical outcome studies using the same measure [13,14,15].

### 3.3. Implementation Measures

Most participants expressed interest in keeping the 6500 K architectural lighting (86.7%) [7]. There were no differences for either group in perceived brightness, comfort, or reported symptoms across conditions (all *p* > 0.14). Reported satisfaction with the architectural light was significantly higher on a scale of 1–5 (1 = very unsatisfied, 3 = neutral, 5 = very satisfied) for the intervention versus baseline in night workers (*p* < 0.05; Table 1), but not day workers (*p* = 0.35). The department elected to keep the 6500 K architectural lighting and timers.

Optional tools were used to some degree by most participants (89%). Of those, most individuals reported tools were easy to use and that they were likely to continue usage after the study (Table 1). Departmental leadership decided against lightbox usage after the study due to a lack of available, dedicated space. Participants were allowed to keep the other two optional components. Additionally, participants reported that the text message reminders, both to use the optional components and to aid in data collection, were helpful (Table S3).

Interviews were conducted with five study participants and two department leaders. Results identified characteristics of the intervention, participants, and inner-organizational setting that could affect implementation. Most notably, participants felt that the intervention was simple to follow and were satisfied with their experiences; however, both participants and department leaders expressed concern that lightbox usage could disrupt patient care and/or was difficult to use because of the inconvenient location. Additionally, both participants and leaders thought that receiving the results of the study would increase the likelihood of future participation; several indicated that medical professionals would be more inclined to participate in the future if the intervention was marketed as “evidence based.” Department leaders indicated that the intervention aligned with staff values but expressed uncertainty regarding how to purchase the equipment required to continue the intervention. Interestingly, when asked why they participated, respondents focused on potential benefits to sleep and well-being, and none mentioned job performance or patient care.

## 4. Discussion

This multi-component, customizable lighting intervention was well received and demonstrated selective improvements under real-world conditions. Primary study strengths are the hybrid effectiveness–implementation design [4], as well as the passive nature of the primary component (overhead lights). Additional strengths include the examination of day workers, a historically-overlooked group in shiftwork intervention studies, the within-subjects and within-schedule control, and the use of text messaging to improve compliance/adherence. While a placebo effect cannot be ruled out, the number of effects over different outcomes, including objective measures such as the PVT, make this unlikely. While night workers rated higher intervention satisfaction and greater likelihood of continued use than day workers, the intervention appeared to benefit day workers more than night. This might reflect the relatively longer exposure to the architectural lighting and/or lightbox, more individual variability in night workers, or both. Unfortunately, we cannot tease out the relative contribution of the multiple components in this pilot study, and future work should aim to do so. Additionally, day workers should not be overlooked as a study population of interest, as they appeared to benefit from this intervention. This is supported by emerging evidence elsewhere that shiftwork creates challenges for day workers [17,18,19].

A large body of research examining the effects of multi-component lighting interventions in simulated nightwork shows great promise (reviewed in [8]). Fewer studies have combined multiple tools to modulate light exposure patterns in field studies, though those that do indicate promise, as well [20,21,22,23,24,25]. Research from numerous fields indicates that the use of multiple intervention strategies may enhance the efficacy of behavioral interventions, including in workplace settings [26,27,28,29]. To our knowledge, this is the first hybrid effectiveness–implementation study using a multi-component approach [5].

This study has several limitations. First, while we consider the multi-component aspect of our study to be a strength, coupled with our relatively small sample size, it has resulted in the inability to tease out individual components of the study (e.g., the effect of blueblockers alone). Additionally, some results seem to be specific (only one time of day, etc.), which may also be a result of our sample size, as well as the difficulty in teasing out relative contributions of time awake, time on shift, circadian time, and effects of sleep loss. Finally, we could have benefited from a de-masked measure of circadian phase as well as higher frequency sampling, though the decision not to do so was made so as to limit participant burden. Such trade-offs are unfortunately often necessary in applied work in shiftworking populations. Future studies with larger sampling sizes that are able to parse out the presumably additive effects of each optional component would be ideal. Furthermore, studies utilizing dynamic lighting sources and measures of circadian phase could potentially differentiate alerting effects of light from phase-shifting effects. Additionally, in the future, more individualized multi-component strategies could potentially account for personal sleep strategies and patterns of alertness.

## 5. Conclusions

Utilizing a hybrid effectiveness–implementation approach [5,6], assessing implementation factors alongside efficacy, we found that a multi-component lighting intervention (1) was met with high rates of satisfaction, ease of use, and likelihood of continued use by both day workers and night workers, (2) improved quality of life in all workers, and (3) selectively improved performance and self-reported sleep in day workers, and alertness on days off in night workers. Hybrid effectiveness–implementation studies take a novel approach that the National Institutes of Health and the Sleep Research Society have jointly suggested be integrated into circadian studies [4]. In short, utilizing multiple components, allowing customizable interventions, and capitalizing on multiple mechanisms of light may be an effective and feasible way of improving the health and performance of shift workers.

## Figures and Tables

**Table 1:** Table of self-reported effectiveness and implementation measures.

	Day			Night		
	Baseline (SEM)	Intervention (SEM)	*p* Value	Baseline (SEM)	Intervention (SEM)	*p* Value
Sleep Quality	3.22 (0.16)	3.80 (0.16)	<0.001	3.30 (0.16)	3.12 (0.16)	0.33
Total Sleep Time (m)	416.03 (32.27)	421.29 (32.23)	0.82	401.29 (25.90)	380.48 (26.16)	0.44
Sleep Onset Latency (m)	31.29 (11.80)	26.91 (11.84)	0.59	23.34 (5.85)	16.08 (6.05)	0.37
WASO (m)	9.10 (2.47)	6.55 (2.54)	0.23	22.08 (6.39)	14.39 (6.40)	0.18
Caffeine (drinks/day)	1.57 (0.25)	1.26 (0.25)	<0.05	1.05 (0.17)	0.96 (0.17)	0.55
Overall QOL	20.01 (1.46)	22.48 (1.69)	<0.05	22.13 (1.10)	23.36 (1.01)	<0.05
Health QOL	20.07 (1.52)	22.79 (1.88)	<0.05	20.30 (1.24)	22.01 (0.98)	<0.05
Family QOL	20.09 (2.18)	22.21 (2.03)	<0.05	24.19 (1.02)	24.25 (1.22)	0.96
Socio-Economic QOL	21.08 (1.37)	22.62 (1.89)	0.16	24.06 (1.29)	24.98 (1.13)	0.17
Psych-Spiritual QOL	18.65 (2.16)	21.77 (1.80)	0.10	22.27 (1.88)	23.58 (1.53)	0.10
Lighting Satisfaction	2.78 (0.22)	3.13 (0.28)	0.35	3.50 (0.53)	4.91 (0.58)	<0.05
Ease of Use	Somewhat—Very Easy	Somewhat—Very Difficult	No Opinion	Somewhat—Very Easy	Somewhat—Very Difficult	No Opinion
Lightbox	80.0%	20.0%	0%	77.8%	22.2%	0%
Eyemasks	83.3%	0%	16.7%	87.5%	12.5%	0%
Blue-blockers	100.0%	0%	0%	100.0%	0%	0%
Future Use	Somewhat—Very Likely	Somewhat—Very Unlikely	No Opinion	Somewhat—Very Likely	Somewhat—Very Unlikely	No Opinion
Lightbox	44.4%	44.4%	11.1%	80.0%	10.0%	10.0%
Eyemasks	55.5%	33.3%	11.1%	60.0%	40.0%	0%
Blue-blockers	55.5%	33.3%	11.1%	80.0%	10.0%	10.0%

SEM = standard error of the mean; m = minutes; WASO = wake after sleep onset; QOL = quality of life; sleep quality and lighting satisfaction are on a scale of 1–5, with 5 being highest; QOL is on a scale of 0–30.

## Supplementary Materials

The following are available online at https://www.mdpi.com/1660-4601/17/23/9141/s1, Figure S1: Spectral power distribution of overhead lights pre- and post- intervention, Figure S2: Vector plot of timing and duration of lightbox use during the intervention week, Figure S3: Actogram of a nightshift worker, Figure S4: Weekly averaged light profiles from Actiwatch data, Figure S5: Hormone profiles for waking hours pre- and post-intervention, Table S1: Participant characteristics, Table S2: Actigraphy-based sleep measures, Table S3: Perceptions about study participation.

## Disclaimer

CDR Yablonsky is a military service member. This work was prepared as part of her official duties. Title 17, U.S.C. §105 provides that copyright protection under this title is not available for any work of the U.S. Government. Title 17, U.S.C. §101 defines a U.S. Government work as work prepared by a military service member or employee of the U.S. Government as part of that person’s official duties. Report #20-37 was supported by the TriService Nursing Research Program grant #N16-503 under work unit no. N1618. The views expressed in this research are those of the authors and do not necessarily reflect the official policy or position of the Department of the Navy, Department of Defense, nor the U.S. Government. The study protocol was approved by the Naval Medical Center San Diego Institutional Review Board in compliance with all applicable Federal regulations governing the protection of human subjects. Research data were derived from an approved Naval Medical Center San Diego, Institutional Review Board protocol number NMCSD.2012.0002.
