# Supplementary material for: A Hybrid Effectiveness-Implementation Study of a Multi-Component Lighting Intervention for Hospital Shift Workers
Source: Int J Environ Res Public Health. 2020 Dec 7;17(23):9141. doi: 10.3390/ijerph17239141 (PMC7730086; doi:10.3390/ijerph17239141)
Supplement: Supplementary file 1 [file ijerph-17-09141-s001.pdf]

International Journal of
Environmental Research and Public Health

MDPI

Supplemental Material for:

A hybrid effectiveness-implementation study of a multi-component lighting intervention for hospital shiftworkers—Harrison et al., 2020

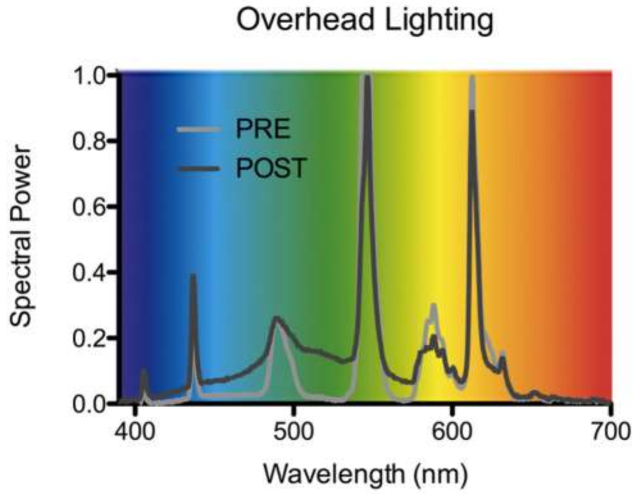

Figure S1. Spectral power distribution of overhead lights pre- and post-intervention. A version of this figure was published in Schmied et al. 2019.

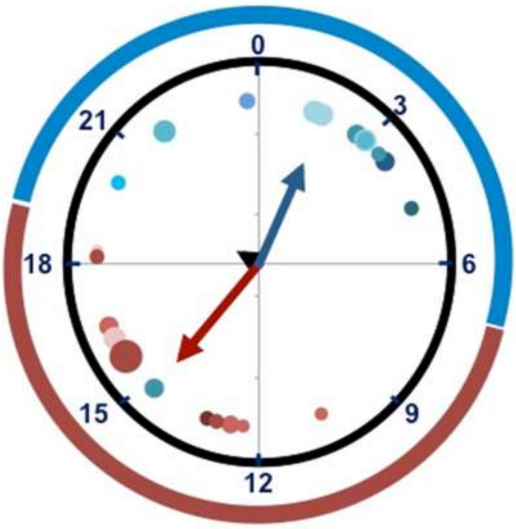

Figure S2. Vector plot of timing and duration of light box use during the intervention week. Each dot represents one session for one participant. Blue dots represent night workers, and red, day. Each individual is represented by a single shade of blue or red. The size of the dot reflects relative exposure length, and the location of the dot around the circle represents the time of day. The position of the red arrow represents the mean exposure time for day workers, and the blue arrow, night workers. The length of the arrow is a function of how clustered the timing is for each group. The black arrow (appears as a triangle) reflects the mean for all participants.

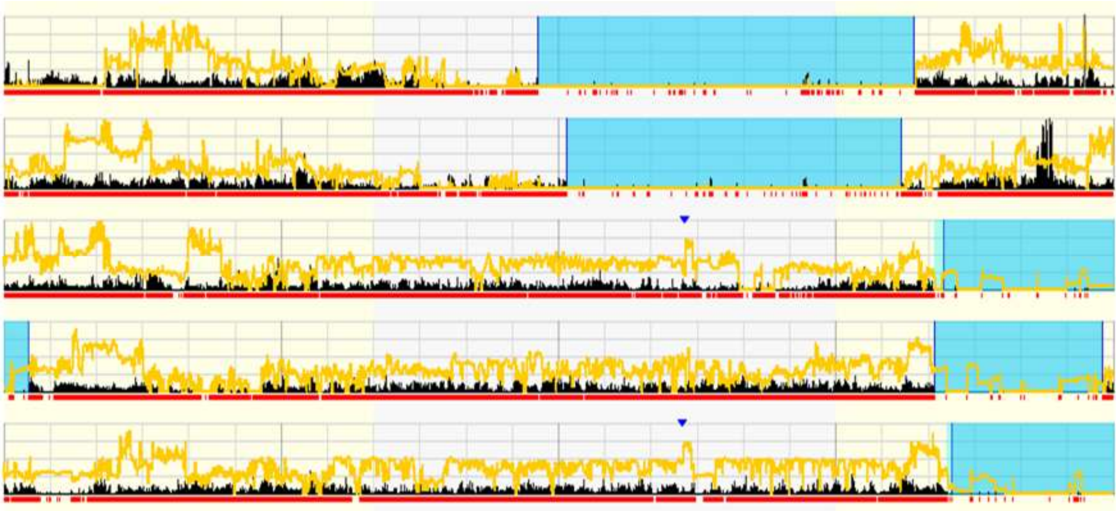

Figure S3. Actogram of a nightshift worker using the light box on-shift. Mid-line of the actogram represents midnight. The two blue triangles indicate times when the participant sat in front of the light box while on shift. In addition to the lightbox usage log, participants were instructed to use the Actiwatch activity markers to track light box use. Note that the light intensity increases at that time.

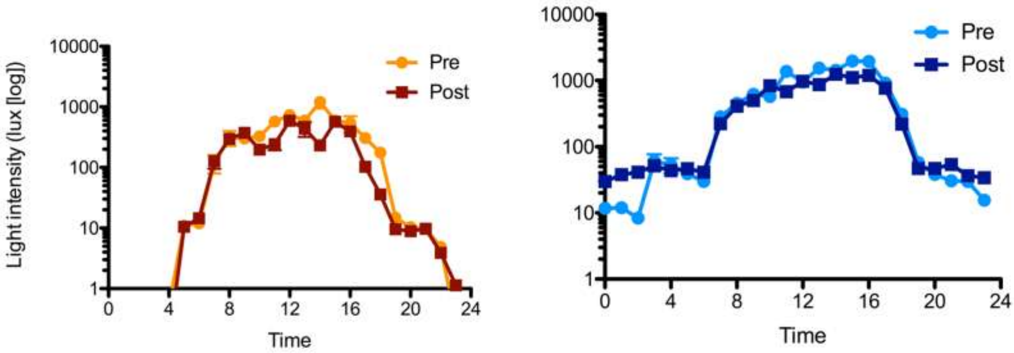

Figure S4. Weekly averaged light profiles from Actiwatch data for both conditions for day (n=7) and night workers (n=10). Light is represented in log units. Values under 1 lux are not shown as Actiwatch-2 models are not sensitive to light in that range per the manufacturer's website. Apparent condition differences in the night group may reflect averaged data, which includes using the relatively high-intensity light box when the ward would otherwise be only dimly illuminated (see Fig S3).

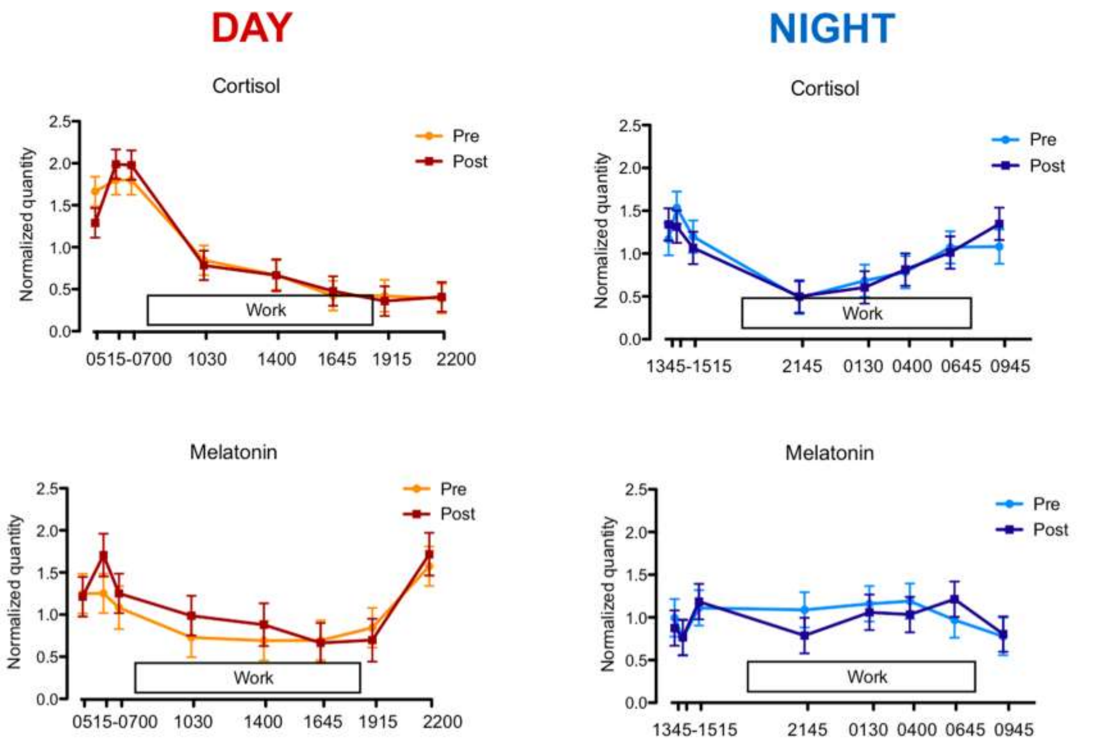

Figure S5. Hormone profiles for waking hours pre- and post-intervention. Cortisol and melatonin were collected via self-administered salivary swabs across a single working day in both conditions. The first three samples were to be completed within the first hour of waking (~wake, wake + 30m, and wake +60 min). Four samples were taken over the course of the work shift (3, 6, 9 and 12 h into the shift). Finally, one sample was taken before the main sleep period, or bedtime, following the work shift (BT). Participants were prompted with text message reminders to collect the samples.

Table S1. Participant characteristics.

	(Mean (SD))
Age, y	*34.74 (8.09)*
History of shiftwork, y	*10.43 (9.14)*
	N (%)
Gender: *Female*	*19 (100.0)*
Nurse (RN)	*16 (84.2)*
Corpsman	*3 (15.8)*
Active duty	*10 (52.6)*
Civilian	*9 (47.4)*
White	*7 (36.8)*
Asian	*2 (10.5)*
Hispanic	*4 (21.1)*
Black	*4 (21.1)*
Other/Multiple	*2 (10.5)*
Single	*3 (15.8)*
Separated/Divorced/Widowed	*4 (21.1)*
Married/Living with Partner	*12 (63.2)*
Sleep and/or Circadian Education:	
None whatsoever	*15 (78.9)*
A few hours or so (e.g. one class)	*2 (10.5)*
A full course	*0 (0.0)*
Other	*0 (0.0)*
Missing/blank	*2 (10.5)*

Table S2. Actigraphy-based sleep measures.

	Baseline	Intervention	
**Day (n=9); (mean±SEM)**			
SE	87.45±1.07	88.60±1.38	p=0.36
TST (m)	408.09±23.28	417.16±11.29	p=0.63
SOL	9.21±1.81	7.94±1.40	p=0.55
WASO	43.84±5.56	47.51±6.20	p=0.49
**Night (n=10); (mean±SEM)**			
SE	85.41±1.10	87.20±1.45	p=0.12
TST (m)	339.08±25.07	331.15±23.30	p=0.80
SOL	6.85±1.05	5.50±1.28	p=0.36
WASO	46.38±4.46	45.79±7.77	p=0.93

Table S2. Between 5-7 days of actigraphy was collected from each subject for each phase of the intervention. Participants wore one of two Actiwatch models (Actiwatch 2 [n=15] and Spectrums [n=4]; Philips Respironics, Murraysville, PA) set to record in 30 sec epochs, and records were scored on the affiliated software (ActiWare v 6.0.9).

Table S3. Perceptions about study participation.

Ranked ease of data collection (easiest-hardest)	
Actigraphy	
KSS	
PVT	
Single Questionnaire (~25 m)	
Sleep Diary	
Saliva Sampling (8/d, 1d/wk)	
Weekly Questionnaires (~10 m)	
Ratings for text message reminders	
For measures:	
Very helpful	*16 (84.2)*
Somewhat helpful	*2 (10.5)*
Not helpful	*0 (0.0)*
Missing	*1 (5.3)*
For intervention:	
Very helpful	*10 (52.6)*
Somewhat helpful	*2 (10.5)*
Not helpful	*0 (0.0)*
Missing	*7 (36.8)*

* **Note:** The longer survey was likely perceived as less burdensome than the shorter, weekly ones because it was not time sensitive and could therefore be completed when convenient.
